# Physical field processing in surimi gelation: From molecular triad modulation to customized nutrition^☆^

**DOI:** 10.1016/j.ultsonch.2026.107892

**Published:** 2026-05-19

**Authors:** Xia Gao, Meng Gui, Liang Gao, Jie Huang, Xiaoqing Ren, Ru Liu

**Affiliations:** a Fisheries Science Institute, Beijing Academy of Agriculture and Forestry Sciences, National R&D Branch Center for Conventional Freshwater Fish Processing (Beijing), Beijing 100068, PR China; b College of Food Science and Bioengineering, Tianjin Agricultural University, National R&D Branch Center for Conventional Freshwater Fish Processing (Tianjin), Tianjin 300392, PR China; c College of Food Science and Technology, Huazhong Agricultural University, National R&D Branch Center for Conventional Freshwater Fish Processing (Wuhan), Wuhan, Hubei 430070, PR China

**Keywords:** Highintensity ultrasound, Acoustic cavitation, Surimi gelation, Protein conformation, Enzyme activity regulation, Personalized nutrition

## Abstract

Surimi-based products are widely consumed due to their huge nutritional values. Especially towards global aging, specific demographics (e.g. the elderly and hypertension patients) have increasingly diverse demands for surimi-based products in terms of texture and nutrition. The gelation properties fundamentally determine the quality attributes of these products. Conventional water-bath heating used to produce surimi-based products relies on conductive heating, which faces inherent limitations in precise temperature control and energy efficiency. To further improve the gelation properties, emerging physical field technologies offer promising alternatives. However, there are few reviews that systematically elucidate and compare the mechanism behind the enhanced surimi gelation properties under different physical field technologies, and further explore how these technologies can be precisely modulated to meet the personalized needs of special demographics. Based on surimi gelation process and principles of physical field technologies, this study systematically elucidated the regulatory mechanisms of these technologies from a molecular perspective, focusing on myosin behavior, transglutaminase activity, and protease inhibition. Subsequently, it further explored how this precise controllability can be harnessed to develop function-specific surimi-based products tailored to distinct consumer groups. The present work showed that physical field technologies enhanced surimi gelation by promoting myosin dispersion/unfolding, strengthening hydrophobic interactions and disulfide bonds, activating transglutaminase, and inhibiting proteases. More importantly, their controllable energy delivery provided a unique platform for creating customized surimi products. Future research should focus on multi-physical field coupling, intelligence processing, and personalized surimi-based foods for specific demographics, thereby advancing the industry toward precision nutrition and sustainable manufacturing.

## Highlights

• This review systematically compared HIU, HPP, and MW in modulating surimi gelation.
• Physical field processing changed myosin conformation and critical enzyme activity.
• Physical field enabled the production of customized foods for special demographics.

## Graphical abstract

**Figure:**
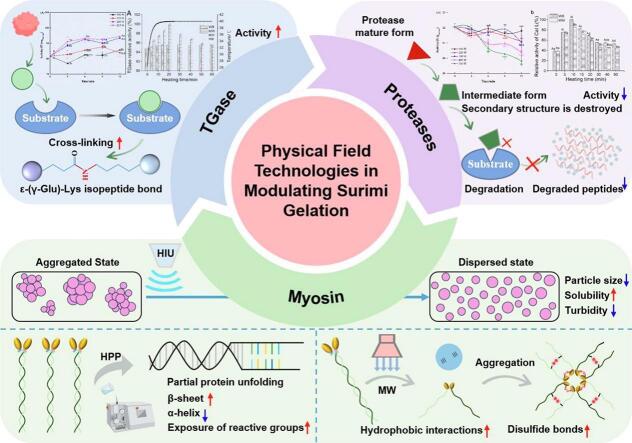

## 1 Introduction

China is the largest aquaculture producer of the world, accounting for more than half of global aquaculture output [1]. Surimi serves as a fundamental raw material for a wide variety of products, such as fish sausage, fish balls, and kamaboko. Owing to its high protein content, low fat content, and ease of preparation, surimi-based products are popular among consumers [2]. The gelation properties are critical indicators for quality evaluation, providing the desired elastic and moist mouthfeel through a stable three-dimensional protein network [3]. Therefore, improving surimi gelation has traditionally been essential for achieving desirable sensory properties, product innovation, and sustainability. However, demographic shifts are reshaping consumer demands for surimi-based products. The global aging population has created a pressing need for senior-friendly foods that are easy to chew and swallow, yet nutritious and palatable [4]. Moreover, the prevalence of lifestyle-related conditions such as hypertension has intensified the demand for low-salt surimi products without compromising sensory quality. These emerging needs call for processing technologies capable of precision modulation, moving beyond the traditional one-size-fits-all manufacturing paradigm toward customized surimi-based products design.

Traditionally, two-stage water bath heating has been employed to produce surimi products, aiming to form an ordered three-dimensional network structure. However, this conventional heating method suffers from inherent limitations, including imprecise temperature control and high energy consumption. Moreover, the *modori* phenomenon – gel weakening caused by endogenous proteases active at 50–70 °C – remains a persistent challenge. To overcome these limitations, physical field technologies – such as high intensity ultrasound (HIU), high pressure processing (HPP), and microwave (MW) heating have attracted increasing attention in recent years [5], [6], [7]. These innovative technologies can directly modulate protein conformation, enzyme activity, and intermolecular interactions through unique energy delivery modes (e.g., cavitation, hydrostatic pressure, electromagnetic fields) [8], [9], [10]. This offers a novel pathway toward precise, efficient, and environmentally friendly processing of surimi gels. Specifically, physical field processing enables rapid traversal of the protease-sensitive degradation temperature zone (50–70 °C). For instance, MW heating achieves rapid volumetric heating, thereby effectively suppressing gel degradation [5], [11]. Meanwhile, non-thermal technologies like HIU can promote the dissolution and dispersion of myosin in low-salt environments, laying a technical foundation for developing healthier, salt-reduced surimi products [8]. Moreover, these technologies typically offer advantages such as low energy consumption, shorter processing times, and ease of integration, aligning with the goals of energy conservation, emission reduction, and sustainable development in the food industry [12].

Given these advantages, several reviews have focused on physical field processing technologies [13], [14], [15], [16], [17]. These reviews have predominantly elucidated the principles and applications of individual technologies (e.g. MW) [13], addressed specific challenges (e.g. low-salt surimi gelation) [14], or examined particular molecular issues (e.g. protein oxidation) [17]. The surimi gelation is governed by three key factors – myosin, endogenous transglutaminase (TGase) and protease. Therefore, it is of great significance to elucidate how physical field technologies modulate surimi gelation from the perspective of these key factors. However, a systematic and integrated comparison of multiple physical field technologies targeting these key factors remains lacking, and the potential to leverage this precise controllability to meet the personalized needs of specific consumer populations has been scarcely explored.

Therefore, this review initially elucidates the molecular mechanism of surimi gelation and the principles of various physical field technologies. Based on this, it then systematically compares the regulatory mechanisms of these technologies from the perspective of three key factors: myosin conformation, TGase activation, and protease inhibition. It further analyzes how these molecular regulations translate into improvements in macroscopic gel properties, and subsequently explores how this precise controllability can be harnessed to develop function-specific surimi-based products tailored to distinct consumer groups. Finally, future research directions toward multi-physical field coupling, intelligence processing, and personalized nutrition are proposed, aiming to advance surimi processing from traditional quality enhancement to the rational design of precision nutrition foods.

## 2 Fundamental mechanism of surimi gelation

### 2.1 Heat-induced gelation process of surimi

**Fig. 1:**
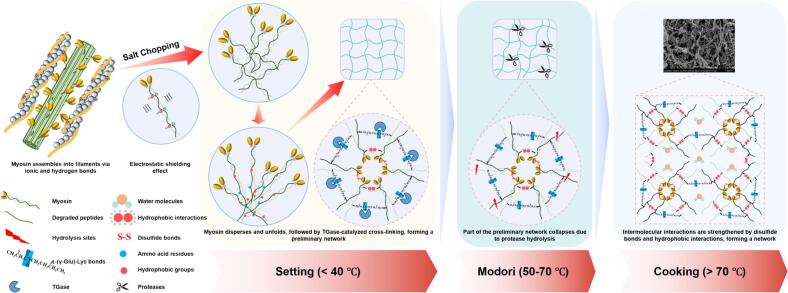
The mechanism of heat-induced surimi gelation.

Surimi is primarily composed of myofibrillar proteins, which accounted for 65%-75% of its total protein content, with myosin being the most critical component [18]. The heat-induced gelation of surimi is a complex process driven by physicochemical interactions, and influenced by key factors including the properties of salt-soluble myosin, endogenous TGase and protease activity [19] (Fig. 1). This process is highly temperature-dependent and primarily involves the unfolding and aggregation of myosin. During the low-temperature setting stage (approximately 4–40 °C), mild heating induces the partial unfolding of myosin, leading to the exposure of active groups, particularly in the rod region, and a conformational shift from α-helix to β-sheet or random coil structures [3]. Simultaneously, endogenous TGase is activated. It catalyzes an acyl transfer reaction between the ε-amino group of lysine residues and the γ-carboxamide group of glutamine residues in the myosin heavy chain (MHC), resulting in the formation of ε-(γ-Glu)-Lys isopeptide bonds. These linkages promote myosin aggregation and the establishment of a preliminary three-dimensional network [20]. Strategies aimed at enhancing myosin unfolding or increasing TGase activity could improve gel properties at this stage. However, a major challenge during gelation is the *modori* phenomenon, which typically occurs between 50–70 °C. Within this temperature range, endogenous proteases exhibit high activity and degrade myosin, leading to the disruption of the preliminary gel network [21], [22]. To mitigate *modori*, common approaches include inhibiting protease activity or rapidly passing the critical temperature zone through controlled heating [13], [22]. When the temperature exceeds 70 °C, surimi enters the cooking stage where myosin further aggregates, primarily through head-head interactions, reinforcing the gel matrix into a stable, ordered three-dimensional network [3], [23]. This step significantly enhances the gel strength and water-holding capacity of the final product. The sol–gel transition during surimi gelation is an irreversible process, culminating in a structural protein gel [24].

### 2.2 Key molecular interactions in network formation

#### 2.2.1 Hydrogen bonds and ionic bonds

Hydrogen bonds form between polar groups, such as the carbonyl group (−C=O) and the amino group (–NH), of protein molecules [25]. Although hydrogen bonds are low in energy and can be easily disrupted by heat, they are deemed to be the most crucial forces involved in the stabilization of protein secondary structures [26]. Hydrogen bonds influence surimi gelation by modulating protein conformation. Specifically, the rearrangement of hydrogen bonds facilitates the transformation of α-helix to β-sheet structures, and the content of β-sheet is positively correlated with the number of hydrogen bonds [27], [28]. Notably, a large number of hydrogen bonds form during the cooling stage, which connect protein molecules, facilitating the formation of a stable three-dimensional gel network and enhancing water-protein interactions within the gel [26], [29]. The formation and stability of hydrogen bonds are influenced by various factors, such as salt concentration, pH, exogenous additives, and processing methods [25], [27], [30], [31]. For instance, the addition of salt facilitates the transformation of α-helix to β-sheet structures in proteins, thereby increasing the number of hydrogen bonds [27]. Besides, Wang et al. [31] reported that shifting pH from 3.0 to 9.0 increased the hydrogen bonds in surimi gels. However, our previous study demonstrated that HIU disrupted hydrogen bonds in surimi gels with 1% NaCl, which might originate from the localized high temperatures generated during cavitation [30]. Thus, modulating hydrogen bonds is of great significance for the gel properties of surimi-based products.

Ionic interactions form through the attraction between oppositely charged amino acid residues (e.g., Arg/Lys/His vs. Asp/Glu), which is responsible for maintaining the native conformation of myosin [32]. External factors such as salt, heat, and HPP can disrupt ionic bonds [33]. Generally, in the low salt environment, myosin molecules aggregate via ionic bonds between charged side-chain residues, leading to filament formation and decreased solubility [34]. Increasing salt concentration (e.g., Cl^-^, Na^+^) shields the charges on the myosin surface, reducing electrostatic repulsion and resulting in myosin dispersion and dissolution [35]. This solubilization is a prerequisite for surimi gelation and is crucial for subsequent network formation. Zhang et al. [7] reported that the combination of MW and ultrasound broke ionic bonds due to enhanced protein dissociation and hydration under the MW field and cavitation. In contrast, some exogenous additives, such as walnut protein isolate, walnut meal and walnut peptide, can strengthen the ionic bonds in sturgeon surimi [36].

#### 2.2.2 Hydrophobic interactions

Hydrophobic interactions play a main role in the formation of the surimi gel network and are strongly heat-driven [37]. At the setting stage, myosin unfolds, exposing initially buried hydrophobic amino acids. Myosin then aggregates through hydrophobic interactions to minimize contact with water, resulting in the preliminary formation of a reversible network. Upon heating to the cooking stage, myosin undergoes sufficient unfolding with extensive exposure of hydrophobic residues. This leads to intense hydrophobic interactions, which become the dominant force maintaining the gel network [20]. The exposure of hydrophobic domains is considered a prerequisite for the formation of myosin aggregates [38]. Exogeneous additives, such as emulsion and fish oil, can alter protein conformation in surimi and facilitate the exposure of hydrophobic groups, thereby strengthening hydrophobic interactions [27], [39]. Furthermore, the physical field of HPP can increase the exposure of buried hydrophobic groups by disrupting the α-helix regions of myosin [40]. However, excessive pressure (e.g., 600 MPa) might promote myosin reaggregation, leading to a decline in exposed hydrophobic groups [40]. HIU has also been reported to induce the exposure of hydrophobic groups [30]. Similarly, the electric field of MW heating can alter myosin structure, thereby exposing hydrophobic groups [41].

#### 2.2.3 Disulfide bonds

Disulfide bonds (–S–S–) are covalent bonds formed by the oxidation of sulfhydryl groups (–SH) from two adjacent cysteine residues. They exist both within and between protein molecules and play a key role in surimi gelation and network formation [42], [43]. –SH groups are considered among the most active functional groups in proteins and are easily oxidized to form disulfide bonds under heating, freezing, or physical field processing [44]. For example, increasing the temperature from 30 to 80 °C initially facilitates protein unfolding and –SH exposure, subsequently strengthening disulfide bonds in myofibrillar protein gels [45]. The addition of oil can enhance head-head and tail–tail interactions of myosin, increasing disulfide bond formation in surimi gels [46]. Moreover, HIU promotes the oxidation of –SH groups to disulfide bonds due to the production of free radicals (·OH and H·) during cavitation [30]. Similarly, pulsed electric field treatment has been reported to generate reactive radicals, promoting the oxidative conversion of exposed –SH groups and facilitating disulfide bridge formation [43]. The combination of HIU and MW treatment has been shown to facilitate the most extensive formation of disulfide bonds [7].

#### 2.2.4 Non-disulfide covalent bonds

Non-disulfide covalent bonds represent a specific category of cross-links, distinct from disulfide bonds, form through catalysis by endogenous TGase in surimi or microbial transglutaminase (MTGase) [47]. During surimi gelation, endogenous TGase catalyze cross-linking between glutamine and lysine residues, forming these non-disulfide covalent bonds, which are specifically referred as ε-(γ-Glu)-Lys isopeptide bonds [30]. At relatively low temperatures, ε-(γ-Glu)-Lys isopeptide bonds can still form due to the existence of endogenous TGase, albeit at a slow rate [48]. As the temperature increases to around 40 °C, myosin unfolds, exposing active residues and providing more substrate for TGase action. Concurrently, this temperature is optimal for TGase activity, leading to the rapid formation of large amounts of ε-(γ-Glu)-Lys isopeptide bonds [47]. The formation of these stable bonds contributes to the establishment of a preliminary gel network, which is resistant to subsequent heating and protease hydrolysis during the *modori* stage, thereby contributing significantly to the ultimate gel strength. Consequently, strategies aimed at strengthening non-disulfide covalent cross-linking – such as using TGase activators (additives or processing methods) and optimizing myosin substrate availability – have been employed to enhance surimi gelation [30].

#### 2.2.5 Synergistic action of molecular interactions

Throughout the surimi gelation process, the covalent and non-covalent bonds described above function synergistically. At relatively low temperatures (4 °C), ionic bonds and hydrogen bonds play primary roles in initial myosin aggregates [34]. As the temperature increases to approximately 40 °C, myosin unfolds, exposing active and hydrophobic groups [38]. Meanwhile, endogenous TGase reaches its optical temperature, leading to extensive cross-linking via ε-(γ-Glu)-Lys bonds, which facilitates the formation of the preliminary gel network [30]. Hydrophobic interactions gradually become more prominent, further strengthening the network. Upon heating to 90 °C, myosin undergoes intense unfolding with massive exposure of –SH groups, which are oxidized to form disulfide bonds [44]. This final step reinforces the gel network, culminating in a stable three-dimensional structure. Among these chemical forces, hydrophobic interactions, disulfide bonds and non-disulfide covalent bonds are the main drivers of surimi gelation, while hydrogen bonds and ionic bonds contribute less to the formation of the final three-dimensional network structure [29]. Physical field technologies can strengthen the gel network by precisely regulating these key chemical bonds.

## 3 Overview of physical field technologies and their principles

### 3.1 High intensity ultrasound

**Fig. 2:**
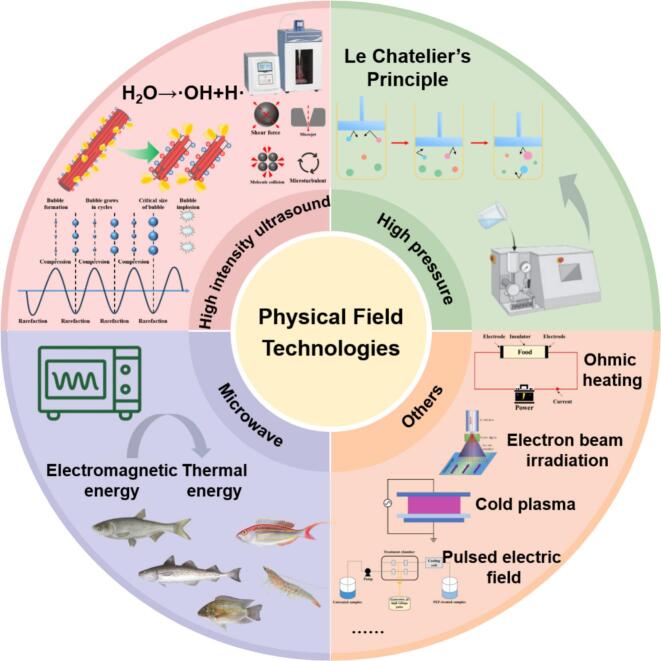
Overview of physical field technologies and their principles.

HIU, typically operating at a frequency of 20–100 kHz and a power intensity of 10–1000 W/cm^2^, has gained widespread attention in food processing due to its advantages of being easy to operate, safe and environmentally friendly [49]. The core mechanism of HIU is acoustic cavitation. When propagating through a liquid medium such as surimi paste, HIU induces the formation, growth, and violent collapse of transient microbubbles over a few acoustic cycles [30]. This collapse generates localized extreme conditions (>5000 K temperature, ∼1000 atm high pressure), accompanied by intense physical effects such as shear forces, micro-jets, and turbulence [50]. Simultaneously, the sonolysis of water molecules produces highly reactive free radicals (e.g., ·OH, H·) [51], which can participate in redox reactions with protein side chains, such as promoting the oxidation of –SH to form −S–S (Fig. 2). However, HIU also has certain disadvantages, including high equipment cost and possible excessive protein oxidation caused by free radicals, since excessive protein oxidation has been reported to deteriorate the color, texture, and water-holding capacity of aquatic products [52].

The combination of physical and chemical effects of HIU significantly modifies protein properties. Physically, the shear forces generated by HIU disrupt protein aggregates, reducing particle size and improving protein solubility and dispersion. More importantly, it induces the unfolding of protein structures, exposing initially buried reactive groups such as hydrophobic groups and –SH groups. This structural pre-conditioning by HIU provides a more favorable foundation for the subsequent heat-induced gelation, priming the proteins for enhanced hydrophobic interactions and disulfide bond formation during heating. Consequently, various studies have demonstrated that HIU pretreatment effectively improves the gel strength of surimi from species such as silver carp, threadfin bream, bay scallop, *Litopenaeus vannamei*, tilapia [30], [53], [54], [55], [56]. It is noteworthy, however, that the extent of improvement is significantly influenced by HIU processing parameters (e.g., intensity, duration, mode) and the matrix properties of the surimi, requiring careful optimization for different applications (Table 1).

**Table 1:** The application of physical field processing in surimi gels.

Physical field processing	Fish species	Ingredients	Processing procedures	Indicators	Reference
HIU	Threadfin bream	NaCl: 0.5%-2%	Samples were sonicated for 30 min with temperature below 10 °C→ 90 °C/30 min, The HIU intensity was 10–16 W/cm^2^	Protein content ↑ Breaking force ↑	[55]
	Silver carp	NaCl: 0–5%	100 kHz, 300 W, 10 min, 20 °C→ 40 °C/1h→ 90 °C/30 min	HIU significantly improved the gel properties of low-salt surimi, but damaged the gel properties of high-salt surimi (4%, 5%)	[30]
	Nemipterus virgatus	NaCl: 1% Curdlan: 0.4%	150 W, 40 kHz, 20 min of ultrasound bath processing → Two-stage water bath heating	Breaking force ↑ WHC ↑	[89]
	Silver carp	NaCl: 1% L-His: 0.1%	The samples were pretreated at 360 W for 10 min→ Conventional two-stage water bath heating	Gel strength ↑ WHC ↑ Cooking loss ↓	[105]
	Silver carp	NaCl: 1%	HIU bath at a frequency of 40 kHz for 0–30 min→ 3D printing → Two-stage water bath heating	The biggest breaking force and WHC of low-salt surimi gels induced by HIU were obtained at 15 min, which was similar to that of high-salt surimi gels	[106]
High pressure	Alaska pollock	NaCl: 0.3%	150, 300 MPa/10 min/10 °C→ 5 °C/24 h	Whiteness ↑ Breaking force ↑	[95]
	Alaska pollock	NaCl: 0.3% MTGase: 0.5% Amino acids: 0.1%	300 MPa/10 min → 5 °C/24 h→ 90 °C/30 min	Breaking force ↑ WHC ↑	[96]
	Threadfin bream	NaCl: 0.5% Polysaccharides: 5%	200 MPa/10 min → 40 °C/30 min → 90 °C/20 min	Gel strength ↑ Expressible water ↓ Whiteness ↑ Microstructures ↑	[60]
	Threadfin bream	NaCl: 0.5% MTGase: 0.1%	150 MPa/15 min → 75 °C/30 min	Whiteness ↑ Textural properties ↑	[59]
	Silver carp	NaCl: 1.5% k-carrageenan: 0.8%	100–500 MPa/10 min → 4 °C/1h→ 35 °C/1h→ 90 °C/30 min	Maximum WHC, gel strength obtained at 300 MPa	[107]
Microwave	Silver carp	NaCl: 1%	40 °C/30 min→ 15 W/g microwave power for 20–80 s	Breaking force ↑ Deformation ↑ WHC ↑	[68]
	Silver carp	NaCl: 1.5%	40 °C/50 min→ 6 kW power at 90 °C holding for 0–20 min	Holding time of 10 min obtained the highest breaking force, deformation and WHC	[69]
	Silver carp	NaCl: 1.7% Lys: 15 mmol/L	40 °C/30 min→ 10 W/g microwave power for 1 min	Gel strength ↑ WHC ↑ Protein degradation ↓	[108]
	Sturgeon	NaCl: 0.3% L-Arg: 0.5%-2%	200 W/1, 2, 3 min	Gel strength ↑ Cooking loss ↓	[71]
	Silver carp	NaCl: 0.3% Arg: 0.5% TGase: 0.3%	5 W/g microwave power for 2, 4, 6 min	The physicochemical properties of low-salt surimi prepared by the combination of microwave and MTGase and Arg were similar to those of high-salt surimi (3%) gels prepared by conventional two-stage heating, especially for 6 min of microwave heating	[109]

Notes: ↑ indicates an increase or improvement, and ↓ indicates a decrease or reduction.

### 3.2 High pressure processing

HPP is a non-thermal technology widely applied in the food industry, involving the treatment of food under hydrostatic pressure, typically ranging from 100 to 1000 MPa [57]. The effects of HPP on proteins obey Le Chatelier principle, whereby an increase in pressure promotes processes that result in a decrease in system volume [58]. Changes in protein hydration volume are also important during HPP (Fig. 2). HPP primarily disrupts the non-covalent bonds (ionic bonds, hydrogen bonds, hydrophobic interactions) that stabilize tertiary or quaternary protein structures, leading to dissociation, unfolding, and even denaturation [59]. In contrast, HPP has minimal effects on covalent bonds, which helps preserve the natural flavor, color, and taste of foods. Furthermore, pressure-induced protein unfolding facilitates subsequent molecular rearrangements during heating, thereby improving gelation properties. HPP offers the advantage of high treatment uniformity, but its widespread application is challenged by high operational costs (Fig. 2).

HPP has been proven to effectively enhance the gelation properties of surimi derived from a wide range of aquatic species, including threadfin bream, tilapia, shrimp, hairtail (*Trichiurus haumela*), *Aristichthys nobilis*, and *Nemipterus virgatus* [6], [60], [61], [62]. Its efficacy is highly dependent on the applied pressure level, with an optimal range typically below 400 MPa. Compared to conventional thermal gels, HPP induces remarkable increases in gel strength (by 38%-40% in hairtail surimi) and water holding capacity (WHC) (up to 27%) [63], [64]. Specifically, HPP promotes the formation of a denser gel network by strengthening hydrophobic interactions and facilitating disulfide bond formation, both crucial for structural integrity [6], [61], [65]. Concurrently, the whiteness of surimi gels is significantly improved, contributing to a more desirable visual appearance [60], [61], [66]. It is noteworthy that the effects of HPP can be influenced by the processing sequence. For instance, applying HPP prior to cooking has been shown to yield superior gel quality compared to applying it after the setting stage, underscoring the importance of process optimization in practical applications [64] (Table 1).

### 3.3 Microwave heating

MW are electromagnetic waves with frequencies ranging from 300 MHz to 300 GHz. The most commonly used frequencies in the food industry are 915 MHz and 2450 MHz [13]. The mechanism of MW processing relies on dielectric heating. Polar molecules, especially water, undergo billions of dipole rotations and rearrangements per second under an oscillating electric field, converting electromagnetic energy into thermal energy. Simultaneously, ions in the food oscillate and migrate within the electric field, synergistically contributing to heat generation (Fig. 2). This mechanism enables MW to penetrate materials and achieve simultaneous internal and external heating, distinctly differing from traditional conduction-based surface-to-core heat transfer [67]. Thus, MW processing offers notable advantages in the food industry, including rapid heating, high energy efficiency, and cleanliness [41]. However, challenges arise from non-uniform heating due to electromagnetic field distribution, sample dielectric properties, and thickness, leading to the coexistence of “cold spots” and “hot spots”.

Surimi, characterized by high moisture and salt content, has a high capacity for dipole polarization and ion conduction, making it highly responsive to MW heating [13]. However, sole MW heating for surimi presents certain drawbacks, primarily non-uniform temperature distribution, which can easily lead to undesirable texture, surface drying, and higher cooking loss [67]. To overcome these limitations, most studies adopt a combination of initial water bath heating followed by MW heating. Using MW heating during the cooking stage helps rapidly traverse the gel degradation zone (50–70 °C), thereby minimizing the *modori* phenomenon [41]. Optimized MW-assisted heating has been documented to effectively enhance the gel strength and WHC of surimi from diverse species, including silver carp, Alaska pollock, shrimp, tilapia, and golden threadfin bream. These improvements arise from enhanced protein interactions – such as hydrophobic interactions, disulfide bonds and hydrogen bonds – under MW irradiation [5], [68], [69]. Besides, synergistic effects between MW and exogenous additives, such as polysaccharides and L-Arg, have also been reported to improve surimi gel properties [70], [71]. Moreover, MW heating can compensate for the inferior gel properties of salt-reduced surimi by intensifying disulfide bonding and hydrophobic interactions. It can also modify the gel microstructure to enhance sodium release during oral processing, contributing to the goal of “salt reduction without taste reduction” [72] (Table 1).

### 3.4 Others

Beyond HIU, HPP, and MW heating, other emerging physical field technologies have also been reported to modify surimi gels, including ohmic heating, electron beam irradiation, cold plasma, and pulsed electric field technology (Fig. 2).

Ohmic heating utilizes the electrical resistance of the food material itself to generate heat volumetrically when an alternating current is passed through it, enabling rapid and uniform internal heating [73]. Its linear heating profile allows for precise temperature control, facilitating rapid passage through the critical gel degradation temperature zone (50–70 °C). This effectively suppresses the activity of endogenous proteases, mitigating the *modori* phenomenon and consequently enhancing gel strength [73]. Studies have demonstrated that ohmic heating (e.g., 10 kHz, 20 V) significantly improves gel strength, WHC, and whiteness of surimi from common carp or Alaska pollock, facilitating the formation of a denser network microstructure [73], [74].

Electron beam irradiation induces chemical modifications in proteins through direct ionization or indirectly via reactive radicals (e.g., ·OH) generated from water radiolysis [75]. At optimal doses (e.g., 5–7 kGy), it effectively promotes the formation of disulfide bonds between myosin molecules and induces a shift in protein secondary structure from α-helix to β-sheet. This results in significant enhancement in gel strength, WHC, and whiteness of surimi from species like *Collichthys lucidus* and hairtail [75], [76]. The effects are notably dose-dependent, with excessively high doses potentially causing protein degradation and quality deterioration.

Cold plasma is generated by ionizing gases, producing a rich mixture of reactive oxygen/nitrogen species, free radicals, and charged particles [77]. These reactive species can attack protein side chains, promoting hydrophobic interactions and disulfide bond formation [78]. When applied to surimi from snakehead, shrimp, or threadfin bream, moderate cold plasma treatment can remarkably increase gel strength, WHC, and elasticity, and contribute to the formation of a denser three-dimensional network [77], [79], [80]. Pulsed electric field (PEF) technology applies short-duration, high-intensity pulses (e.g., 15 kV/cm). It functions primarily through electroporation, altering the state of protein aggregates [43]. Moderate PEF treatment can induce conformational changes in myosin, strengthening hydrogen bonding and hydrophobic interactions, thereby significantly enhancing the gel strength and WHC of surimi gels [43]. However, its efficacy exhibits an intensity-dependent effect.

In summary, these emerging physical field technologies modulate surimi gel properties through diverse pathways, including thermal effects (ohmic heating), radical chemistry (electron beam irradiation, cold plasma), and electromagnetic effects (PEF). The inherent improvement mechanism of these physical field technologies in surimi gelation will be further discussed in the following section.

## 4 Mechanisms of physical field technologies in modulating surimi gelation

### 4.1 Modification of protein conformation and aggregation behavior

Myosin is the most important functional protein in surimi gelation, and its unfolding and aggregation are responsible for the resultant gel properties. Importantly, the well-dispersed state of salt-soluble myosin prior to heating is critical for surimi gelation. Modulation by physical field technologies helps improve gel properties. In its native state, myosin exists as aggregates rather than monomers, held together by weak interactions such as ionic bonds and hydrogen bonds.

**Fig. 3:**
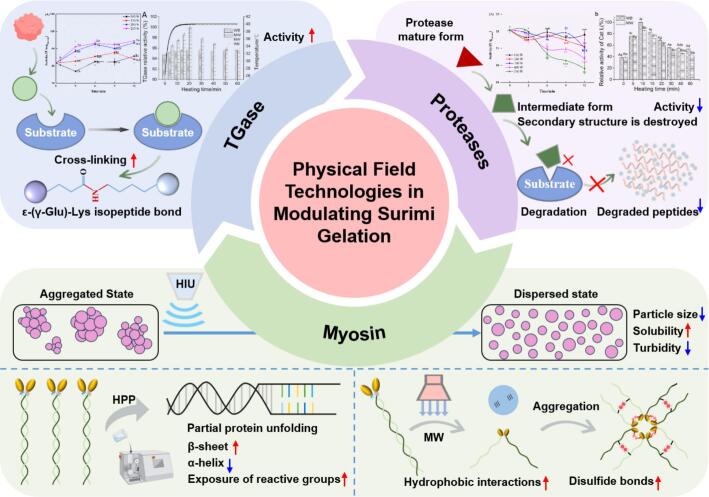
The proposed mechanism of physical field processing in modulating surimi gelation. HIU indicates high intensity ultrasound, HPP indicates high pressure processing, and MW indicates microwave.

HIU disrupts myosin aggregates via shear forces, as evidenced by reduced particle size, increased solubility, and decreased turbidity [8]. Furthermore, HIU causes significant changes in myosin structure, including decreased Ca^2+^-ATPase activity and increased surface hydrophobicity. Meanwhile, HIU facilitates the oxidation of –SH to –S–S– in myosin, a process originating from the free radicals generated during cavitation [49] (Fig. 3).

HPP disrupts hydrogen bonds, resulting in a decrease in α-helix content and a simultaneous increase in β-sheet structures, thereby inducing myosin unfolding [40] (Fig. 3). Subsequently, myosin unfolding induced by these physical field technologies prior to heating facilitates the exposure of buried –SH and hydrophobic groups. Upon subsequent heating, the dispersed myosin further stretches, exposing most of the initially buried reactive groups. Under moderate HPP, the rate of myosin unfolding exceeds that of aggregation. This facilitates head-head interactions mediated by hydrophobic interactions and disulfide bonds, leading to the formation of a three-dimensional honeycomb network [29].

MW heating is often employed to modify myosin aggregation behavior due to its rapid and volumetric heating characteristics. MW irradiation exerts a disruptive effect on myosin molecules, perturbing the electric field distribution and electrostatic interactions between charged residues. This induces changes in the secondary and tertiary structures of myosin. MW heating at elevated temperatures facilitates the formation of hydrophobic interactions and disulfide bonds, thereby promoting myosin aggregation [41] (Fig. 3).

In summary, HIU is usually applied to improve myosin dispersion and solubilization prior to heating, whereas HPP and MW are applied during the gelation process itself to modify myosin aggregation behavior. Thus, all these technologies modulate myosin along a pathway encompassing “dispersion state-conformational changes-aggregation behavior”. However, it is noteworthy that the choice of suitable processing parameters is crucial.

### 4.2 Regulation of transglutaminase

Endogenous TGase is well acknowledged to be responsible for surimi setting by promoting cross-linking between lysine and glutamine residues on MHC, forming ε-(γ-Glu)-Lys isopeptide bonds. These bonds constitute a major force driving for surimi gelation. The catalytic capacity of TGase depends on both its enzymatic activity and substrate accessibility. HIU can enhance the activity of both MTGase and endogenous TGase activity, with a greater extent of improvement observed for MTGase [81]. Spectroscopic analyses (intrinsic fluorescence and UV absorption) suggest that HIU enhances MTGase activity by altering its tertiary structure, resulting in the exposure of reactive –SH groups and aromatic amino acids, without affecting its intrinsic disulfide bonds. Moreover, the catalytic efficiency of TGase on myosin under HIU relies on substrate dispersion. In a low-salt environment (0.1 mol/L), applying HIU to the entire system containing both MTGase and myosin yields the greatest improvement in myosin gelation properties [81]. In contrast, in a high-salt environment (0.6 mol/L), applying HIU to TGase alone prior to mixing with myosin is more beneficial for gelation [82].

MW heating operates through electromagnetic fields that directly induce molecular vibration and thermal effects, potentially leading to conformational changes in enzyme proteins. Cao et al. [83] demonstrated that MW treatment significantly enhanced TGase activity compared to conventional water bath heating at the same rate. After 20 min at 40 °C, MW irradiation increases TGase activity by approximately 4.38%. This enhancement is attributed to MW-induced conformational changes that expose active sites and facilitate substrate binding. However, when TGase is applied in a mixed system with myofibrillar protein, its cross-linking efficiency under MW treatment does not surpass that of water bath heating. This may be due to MW-induced aggregation of myofibrillar proteins and increased formation of disulfide bonds, which create steric hindrance and limit TGase access to glutamine and lysine residues. Furthermore, MW treatment promotes cross-linking predominantly in the head region of myosin, whereas water bath heating favors cross-linking in the tail region, thereby influencing gel network formation efficiency [83]. Thus, while MW treatment can enhance TGase activity in isolation, its catalytic efficacy in complex protein systems is considerably constrained by substrate accessibility and structural alterations. This highlights the importance of considering the synergistic regulation of enzyme activity, protein dispersion, and aggregation behavior when applying MW technology, especially in low-salt surimi systems.

Regarding HPP, the endogenous TGase activity in *Nemipterus virgatus* surimi continuously decreases with increasing pressure due to volume reduction [84], although this effect is less pronounced than with thermal treatment. Moderate pressure below 200 MPa can maintain more than 86% of the original TGase activity, which is considered a main reason for improved gel strength under HPP [85].

### 4.3 Regulation of protease

In addition to TGase, endogenous proteases in surimi are also key factors determining gelation quality, as they cause protein degradation and induce the *modori* phenomenon. Endogenous proteases can be classified into four types based on their catalytic center: cysteine proteases, serine proteases, aspartic proteases, and metalloproteinases. The main protease responsible for *modori* varies among fish species. Our previous study indicated that cysteine proteases are primarily responsible for *modori* in silver carp surimi. HIU can significantly decrease the activity of cathepsin L (Cat L, a cysteine protease), with the degree of inactivation being more pronounced at higher HIU power [86]. The inactivation of Cat L under HIU is considered to be related to its conversion from a mature form to an intermediate form, characterized by reduced particle size and altered tertiary structure, leading to disruption of its active site. Correspondingly, protein degradation is inhibited.

MW treatment significantly inhibits the activity of endogenous proteases, such as Cat L, primarily through its unique electromagnetic field effects and non-thermal mechanisms. On one hand, MW-induced vibration of polar groups and molecular resonance alters the enzyme’s secondary structure, manifested as a significant decrease in α-helix content and an increase in disordered structures, thereby disrupting the conformational stability of its catalytic active center. On the other hand, the MW electric field promotes the cleavage and rearrangement of disulfide bonds within the enzyme molecule, leading to decreased total sulfhydryl content and increased exposed sulfhydryl groups, which further compromises the structural integrity of the active site [87]. Additionally, dynamic light scattering indicates that MW treatment causes Cat L molecules to adopt a more compact conformation, with a reduced ratio of radius of gyration to hydrodynamic radius (Rg/Rh) and increased molecular density. This results in burial of the active center and further impairs substrate-binding capacity. These structural changes collectively decrease protease activity, effectively inhibiting the hydrolysis of myofibrillar proteins – particularly MHC – thereby mitigating the *modori* and enhancing the textural properties and WHC of surimi gels [87].

Qiu et al. [88] reported that HPP from 200 MPa to 500 MPa could reduce the activity of myofibril-bound serine protease (MBSP), with the reduction being more pronounced with increasing pressure and processing time. This is because HPP alters the three-dimensional configuration of MBSP, inducing subtle changes in the active site, which results in loss of enzymatic activity. Furthermore, HPP reduces the proteolysis of myofibrillar protein by MBSP, as evident from preserved MHC bands in electrophoresis, thereby enhancing gel strength [88]. However, the detailed conformational changes underlying protease inactivation under HPP may require further investigation.

## 5 Comparative analysis of physical field technologies processing on surimi gel properties

### 5.1 Textural properties

Textural properties – including breaking force, deformation, hardness, springiness, cohesiveness, gumminess, and chewiness – are among the most important indicators for evaluating surimi gel quality and are significantly influenced by processing methods. The potential of HIU to improve surimi gel properties was early demonstrated by Zhang et al. [56], who investigated the effect of HIU pre-treatment intensity prior to chopping. They found that HIU treatment at 250 W and 100 kHz significantly increased the gel strength of tilapia surimi from approximately 400 g·cm to 700 g·cm. The effect of HIU on surimi gel strength depends on various factors. For instance, applying HIU before salt-chopping significantly increases the breaking force and deformation of silver carp surimi gels to 1007 ± 62.43 g and 14.6 ± 0.16 mm, respectively, compared to the control (723.71 ± 69.22 g, 12.69 ± 0.60 mm). In contrast, HIU applied after salt-chopping or stuffing into PVC casings has only a slight effect on gel properties [50]. As discussed in section 4.1, HIU promotes myosin dispersion prior to heating, which strengthens molecular interactions during gelation process and thereby facilitating the formation of dense microstructures. This contributes to the enhanced textural properties observed in silver carp surimi. However, HIU typically has only slight effect on the springiness of surimi gels.

Chen et al. [84] investigated the effect of HPP from 100 to 600 MPa on the gel strength of *Nemipterus virgatus* surimi and reported that moderate pressure levels (300–400 MPa) yield the optimal gel strength. However, the gel strength of surimi gels prepared by HPP alone is generally below 500 g·cm. Zhou et al. [62] compared various HPP procedures, including HPP alone (300 MPa, 30 min, room temperature) and a combined process of HPP followed by two-stage heating (100 MPa, 5 min, room temperature → 40 °C, 30 min → 90 °C, 20 min). The results showed that surimi gels prepared by the combined HPP and heating process exhibited significantly higher breaking force (793.33 ± 40.72 g) and deformation (14.98 ± 0.02 mm) than those prepared by heating alone (407.36 ± 44.36 g, 10.03 ± 0.17 mm) or HPP alone (586.00 ± 19.49 g, 14.95 ± 0.03 mm). Moreover, the combination of HPP with polysaccharides enhances the gel strength of low-salt (0.5% salt) threadfin bream surimi gels. Interestingly, HPP induces a greater percentage increase in gel strength for low-salt surimi gels compared to their high-salt counterparts [60].

MW heating has been demonstrated as a promising tool for improving surimi gel strength. Studies show that both low (3 W/g) and high (7 W/g) power levels can increase gel strength, with the effect being more pronounced with longer heating times. This is because MW heating promotes more extensive protein cross-linking than conventional water bath heating, resulting in the formation of uniform microstructures and thus improving the breaking force and deformation. Cao et al. [67] investigated replacing the first stage of two-step water bath heating with MW heating and found this substitution detrimental to gel strength. This is because MW heating at 40 °C is not conducive to myosin unfolding and aggregation, leading to reduced gel strength. In contrast, replacing the second heating step with MW significantly improves gel strength, owing to its rapid heating capability which allows quick passage through the critical gel degradation zone (50–70 °C). Furthermore, MW heating has been applied in combination with additives or to salt-reduced surimi systems. The combination of MW heating (80 °C, 2 min) with 0.3% tremella power increased the gel strength of *Nemipterus virgatus* surimi by 96.84% compared to conventional water bath heating, achieving a value of 2314.85 ± 189.67 g·mm [89]. However, excessively long MW heating times can cause protein degradation, leading to a decrease in gel strength [69].

### 5.2 Water holding capacity

WHC reflects the degree of protein-water interactions and is a critical indicator for assessing the quality of surimi gels [90]. Although surimi gels prepared with 2% salt already exhibit high WHC (95.36% ± 0.17), HIU can further enhance it significantly to 95.75% ± 0.14 [30]. Salt plays a crucial role in surimi gelation due to the salt-solubility of myosin. Surimi gels with reduced salt content show markedly lower WHC (85.02% ± 0.11) compared to those with normal salt levels (2%). Under these conditions, HIU can compensate for the inferior properties of low-salt surimi gels, improving WHC to 94.12% ± 0.44 [30]. This enhancement may be attributed to HIU improving myosin dispersion in the low-salt environment and further exposing hydrophilic groups, thereby strengthening protein-water interactions during heating and improving WHC. On the other hand, HIU facilitates the formation of microstructures with smaller pore sizes, which also contributes to trap more water. In addition, combining HIU with exogenous additives can further improve the WHC of low-salt surimi gels. For instance, compared to untreated samples, the combination of HIU and soy protein isolate decreased cooking loss from 52.19% to 28.18% and progressively increased WHC. This phenomenon can be attributed to HIU inducing partial unfolding of myofibrillar protein (MP) and soy protein isolate (SPI), exposing buried hydrophobic and sulfhydryl groups that strengthen intermolecular interactions and trap water molecules [91]. Furthermore, HIU disrupts the particle sizes of MP and SPI, enhancing hydrophilic interactions between protein and water molecules and thus improving water retention.

Ma et al. [92] investigated the effect of HPP from 0.1 to 600 MPa on the WHC of surimi gels and found that moderate pressure (≤400 MPa) significantly improved WHC to 88%, representing a 119% increase compared to the control. Similarly, Huang et al. [85] employed pressures from 0.1 to 300 MPa and showed that increasing pressure raised the WHC of surimi gels from 63% (0.1 MPa) to 87% (300 MPa). This improvement is due to pressure-induced formation of a continuous and compact gel network that effectively trap water. In addition, TGase enhances WHC by catalyzing protein cross-linking, facilitating the formation of dense microstructures that hold more water. Notably, the enhancement of WHC by TGase addition can be more significant under HPP than with heating alone. The combination of 100 MPa HPP with TGase produced the greatest improvement, increasing WHC from 68% (without TGase) to 86%. However, the extent of improvement diminishes at higher pressures: from 84% to 89% at 200 MPa and from 87% to 89% at 300 MPa. This phenomenon originates from the fact that at relatively lower pressures, the protein unfolding rate induced by HPP is slower than the aggregation rate. This favors protein cross-linking catalyzed by TGase, resulting in the formation of a continuous structure that holds more water and increases WHC.

Continuous MW heating improves WHC by 4.74% compared to conventional water bath heating. Moreover, combining MW heating with tremella power (TP) further improves WHC by 6.95%. Since a tighter gel structural matrix generally correlates with stronger WHC, the enhanced WHC under MW and TP is speculated to originate from a denser microstructure [89]. In addition, MW heating has been demonstrated to improve the WHC of low-salt surimi gels (0.5%). Surimi gels prepared by MW heating for 3 min had a WHC of 60.92% (0.5% NaCl), which is lower than that of regular-salt gels prepared by conventional water bath heating. However, when MW heating is combined with 2% L-Arg, the WHC increases to 83.24%, comparable to reported values for regular-salt surimi gels [93]. L-Arg appears to increase the hydration of MP and strengthen protein-water interactions, thereby decreasing the mobility of water molecules within the surimi gels.

## 6 Toward texture modulation and nutrition design for specific demographics

Building upon the enhanced gel properties achieved through physical field processing discussed in Section 5, this section extends the discussion on how these technological capabilities can be strategically leveraged to address the specific needs of different consumer populations. While traditional surimi processing aims for a one-size-fits-all product with maximized gel strength and elasticity, the controllability of physical field technologies opens new avenues for precision manufacturing of surimi products with customized functionalities tailored to specific demographics.

### 6.1 Texture modulation for specific demographics

Different consumer groups have distinct requirements for surimi texture. For instance, elderly individuals and patients with dysphagia require soft, easy-to-swallow foods with high fluidity, whereas hypertensive patients benefit from low-salt products without compromising palatability. Physical field technologies offer versatile tools to address these divergent needs through precise modulation of gel microstructure. Regarding texture softening for elderly-friendly foods, HIU has been proven to tenderize meat by disrupting muscle fibers, suggesting its potential for creating surimi products with reduced hardness while maintaining cohesiveness. Furthermore, physical field technologies such as HIU and HPP can stabilize high internal phase emulsions, which serve as effective texture modifiers for designing dysphagia-oriented foods [4], [94], [95]. These emulsion-based systems can be incorporated into surimi matrices to engineer desirable mouthfeel characteristics for consumers with chewing and swallowing difficulties.

In parallel with texture softening for elderly populations, physical field technologies also offer effective strategies for addressing the dietary needs of hypertensive patients through the production of salt-reduced surimi products. In conventional surimi processing, a high salt concentration (2%–3% NaCl) is typically required to fully extract salt-soluble myosin and obtain high quality gels. However, high salt intake is detrimental to human health, being directly linked to hypertension. The World Health Organization advocates for reduced salt intake, and corresponding national policies aim for a 20% reduction. Consequently, producing salt-reduced surimi products represents a major industry trend. A significant challenge lies in maintaining gel quality while reducing salt content. Physical field processing methods have been widely reported as effective strategies for producing salt-reduced surimi gels with enhanced gel properties. For instance, HIU improves the gelation properties of silver carp surimi with only 1% salt. The improvement mechanism involves HIU disrupting myosin filament structure, promoting myosin dispersion and solubilization in the low-salt environment, and subsequently enhancing protein interactions during heating – a prerequisite for effective gelation. Moreover, HIU activates endogenous TGase, facilitating cross-linking during setting, and inactivates endogenous proteases, protecting proteins from degradation and alleviating gel cracking. These combined contribute to the improved gel properties of low-salt surimi products.

The use of HPP to prepare low-salt surimi gels was early reported by Cando et al. [96] using Alaska pollock surimi with only 0.3% salt. They showed that applying 300 MPa improved the mechanical and sensory properties of reduced-salt gels, achieving values similar to those of gels made with 3% salt. This improvement is closely related to pressure-induced protein denaturation and unfolding. Building on this, Cando et al. [97] further investigated combining HPP with MTGase and amino acids. They found that HPP improved the physicochemical properties of surimi gels, with the combination of HPP, MTGase, and cystine being particularly effective due to enhanced protein cross-linking, as evidenced by reduced MHC band density. HPP has also been combined with polysaccharides to improve the low-salt surimi gel properties. For example, combining 200 MPa HPP with polysaccharides significantly enhanced the gelation properties of low-salt (0.5% salt) threadfin beam surimi, yielding higher gel strength and whiteness. This treatment facilitated a secondary structural shift from α-helix to β-sheets, promoting protein interactions during subsequent heating and leading to the formation of a more compact network microstructure with small voids, thereby reducing expressible water [60].

The potential of MW heating for preparing low-salt surimi gels was explored by Fu et al. [68], who studied the effect of MW heating duration (after water-bath setting) on silver carp surimi with 1% salt. The mechanical and functional properties of low-salt gels are significantly improved by MW heating for 60 s and 80 s, except for cooking loss. Notably, the breaking force of low-salt surimi gels (1%) prepared with 60 s of MW heating (569.7 ± 30.6 g) exceeds that of regular-salt gels (2%) prepared by conventional two-sage water-bath heating (556.8 ± 24.2 g). Moreover, MW heating inhibits protein autolysis during gelling, as indicated by TCA-soluble peptides analysis, verifying its ability to rapidly traverse the gel-cracking zone. Beyond standalone MW heating, combing it with additives also improves low-salt gel properties. For instance, Shi et al. [93] employed MW heating combined with TGase and L-Arg on surimi containing only 0.3% salt, entirely replacing water-bath heating. They found that after 6 min of MW heating, the resulting low-salt gels exhibits physicochemical properties similar to regular-salt (3%) gels produced by conventional two-stage heating, likely due to comparable levels of disulfide bonds, although a slight decrease in whiteness is observed. This approach provides an innovative, time- and energy- efficient method for preparing heathier and more sustainable surimi-based products.

### 6.2 Nutrient design through multi-functional fortification

For the elderly, a single food system is insufficient to meet their diverse nutritional needs. Surimi, as an ideal food matrix, can be fortified with specific nutrients to effectively address the nutritional challenges faced by this population. For example, the addition of Ca^2+^ helps prevent osteoporosis, the incorporation of fish oil rich in DHA benefits cognitive health, and the supplementation of dietary fiber through polysaccharides improves digestive function. Importantly, many of these nutrients play dual roles – simultaneously enhancing nutritional value and contributing to gel structure formation – a synergistic effect that can be amplified by physical field technologies. This section discusses how key nutrients can be incorporated into surimi using physical field technologies to achieve synergistic benefits for both nutrition and gel quality.

#### 6.2.1 Ca^2+^: a dual-functional fortifier

Among various fortification strategies, Ca^2+^ deserves particular attention due to its multifaceted role in the surimi gel system. On one hand, Ca^2+^ acts as salt bridges to enhance intermolecular interactions among proteins, thereby improving physical cross-linking. On the other hand, it can activate endogenous TGase to promote covalent cross-linking, consequently reinforcing the gel network. Importantly, moderate cross-linking does not lead to excessive harness. Therefore, when used at an appropriate concentration, Ca^2+^ serves as a promising candidate for developing senior-friendly surimi products that are both nutritious and texturally suitable.

Physical field technologies can amplify these beneficial effects. An et al. [98] reported that the combination of HIU and Ca^2+^ promotes myosin unfolding, facilitating intermolecular interactions between Ca^2+^ and myosin. Furthermore, HIU reduces the critical concentration of Ca^2+^ required to aggregate myosin, enabling effective gelation with lower Ca^2+^ levels. Similarly, moderate HPP (100–300 MPa) combined with CaCl_2_ enhances myosin gel properties of golden threadfin bream by stabilizing various molecular forces within the gel system, contributing to the formation of a compact and networked myosin/Ca^2+^ composite gel microstructure [99].

#### 6.2.2 Lipids: Balancing nutritional enrichment and gel integrity

Lipids contribute to the physicochemical, flavor, and mouthfeel properties of surimi gels; however, a significant portion is removed during the rinsing process. To compensate for this loss, exogenous lipids are added to enhance the sensory and nutritional properties of surimi gels [100]. However, incorporating olive oil significantly decreases the breaking force and gel strength of surimi gels, with higher oil concentrations (>3%) tending to result in even lower breaking force [46]. A possible explanation is that adding oil dilutes the protein concentration within the network structure, thereby weakening protein interactions. HIU can significantly improve the gel properties of surimi gels containing low oil concentrations (below 3%). At low oil concentrations, HIU promotes the formation of hydrogen bonds and disulfide bonds, facilitating the development of compact, homogenous microstructures with smaller pore diameters, thereby significantly enhancing gel strength and WHC. However, HIU has little effect on improving the poor gelation properties of surimi gels with a high oil concentration (>3%) [46]. The results suggest that the optimal oil concentration should be considered when combined with HIU. Similarly, Jiao et al. [39] reported that adding fish oil damages the textural properties of surimi gels by disrupting the protein matrix, primarily due to obstruction by large oil droplets. Replacing the conventional second heating step with MW heating significantly enhances the gelling properties and WHC of surimi gels, with the enhancement becoming more pronounced as the fish oil content increases. This is because MW heating strengthens oil-protein interactions via non-covalent bonds (ionic bonds, hydrogen bonds, and hydrophobic interactions) and disulfide bonds formed through mild oxidation of the fish oil, leading to the generation of a more compact network. Furthermore, the recommended amount of fish oil for MW processing should not exceed 9% [101].

#### 6.2.3 Polysaccharides: enhancing texture and dietary fiber content

Polysaccharides are widely used as modifiers to enhance gel product performance by influencing protein interactions and promoting cross-linking network formation. They are commonly incorporated to develop surimi products with improved taste, flavor, and nutritional value [102]. Physical field technologies can be combined with polysaccharides to further strengthen the gel properties of surimi gels. He et al. [103] investigated the synergistic improvement effects of HIU and oat β-glucan (OG) on the gel properties of silver carp. OG can induce myosin unfolding and act as a filler in the surimi matrix. The combination with HIU facilitates the dispersion of OG within the surimi matrix and further strengthens myosin-OG interactions via disulfide bonds and hydrophobic interactions. This synergy constructs a more uniform and ordered gel network, contributing to improved gel strength and WHC. Starch, another polysaccharides additive, is also used to enhance surimi properties, primarily as a filler. A recent study by Li et al. [104] demonstrated a more sophisticated synergistic strategy of HPP and starch. They employed HPP to optimize corn starch-myristic acid complexation, yielding V-type crystalline structures with enhanced thermal stability, emulsification, and rheological properties. Furthermore, incorporating 20% starch-myristic acid significantly reinforces shrimp surimi gel networks via starch-protein interactions, improving gel strength and water retention. Similarly, Ji et al. [70] reported that MW treatment enhances the stretching and expansion of konjac glucomannan chains within an Alaska pollock surimi gel network, thereby improving the gelling and WHC of the protein–polysaccharides complex gels.

## 7 Future perspectives

**Fig. 4:**
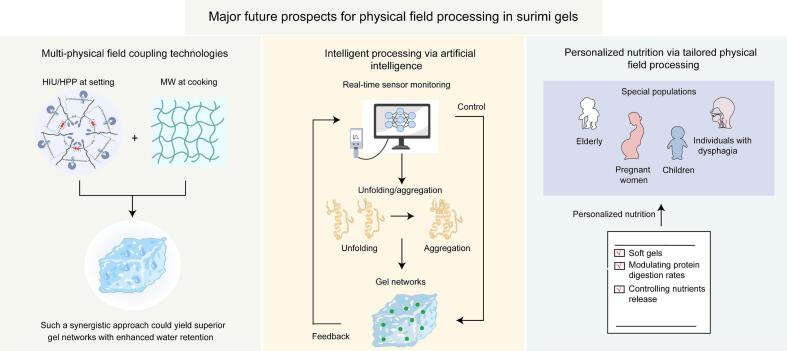
Major future prospects for physical field processing in surimi gels.

Compared to conventional methods, these innovative physical field technologies are more environmentally friendly and energy efficient, contributing significantly to the sustainable development of the surimi industry. However, several challenges and opportunities remain for future research (Fig. 4).

(1) Multi-physical field coupling technologies. The current application of single physical field technology is usually targeted at modifying specific stages of surimi gelation. For instance, HIU is effective at dispersing myosin prior to heating, whereas MW heating is effective at promoting myosin aggregation and is typically applied during the cooking stage. It is hypothesized that strategically combining these technologies, based on their stage-specific maximal effects, could further enhance gel properties. For example, applying HIU or HPP during the setting stage could maximize cross-linking reactions, followed by MW heating during the cooking stage to rapidly traverse the gel cracking zone and promote myosin aggregation. Such a synergistic approach could yield superior gel networks with enhanced water retention and improved energy and time efficiency. However, the synergistic effects of multi-physical field processing on surimi gel properties require further investigation.
(2) Intelligent processing via artificial intelligence. The dynamic changes in myosin and endogenous enzyme behavior under physical field technologies are crucial for gel formation. Further research should explore integrating real-time sensors (e.g., near-infrared spectroscopy, acoustic sensors, or impedance spectroscopy) with machine learning models (e.g., artificial neural networks, random forests, or support vector machines) to monitor and control protein unfolding, aggregation, and gel network formation during physical field processing. These models can correlate spectral or acoustic signatures with gel quality parameters, enabling closed-loop control of processing parameters. Such an intelligent system would facilitate adaptive optimization, reduce batch-to-batch variation, and ultimately achieve smart manufacturing of surimi gels with precise quality control tailored to specific product requirements.
(3) Personalized nutrition via tailored physical field processing. The controllability inherent to physical field technologies lays a foundation for personalized nutrition. Further research should move toward predictive and systematic design of surimi products with tailored functionalities. This includes establishing quantitative relationships between processing parameters and specific textures (e.g., soft gels for elderly with dysphagia), modulating protein digestion rates (fast- or slow-digesting), and controlling the release profiles of specific nutrients. Such advances would enable the rational design of surimi products that meet not only broad demographic needs but also individual physiological requirements, realizing the full vision of precision nutrition.

## 8 Conclusion

The development and consumer acceptance of surimi-based products rely largely on their gelation properties. Innovative physical field technologies offer a promising alternative to conventional thermal methods, enabling more precise, efficient, and sustainable manufacturing. This review provides a comprehensive and integrated analysis of how physical field technologies – particularly HIU, HPP, and MW heating – modulate surimi gelation, with a focus on core determinants: myosin conformational behavior, endogenous TGase activity, and protease inhibition. Crucially, this review moves beyond a simple comparison of technologies to establish a mechanistic framework linking molecular regulation to product functionality. It demonstrates that physical field processing enhances gel properties primarily by: (i) promoting myosin dispersion and unfolding, thereby exposing reactive sites for subsequent cross-linking; (ii) activating endogenous TGase to facilitate ε-(γ-Glu)-Lys isopeptide bond formation; and (iii) inhibiting endogenous proteases (e.g., Cat L, MBSP) to mitigate the *modori* phenomenon. These molecular behaviors translate into improved macroscopic gel properties, including enhanced gel strength and WHC. Importantly, this review extends the discussion from conventional quality improvement to application-driven innovation. It highlights how the precise controllability of physical field technologies can be harnessed to address the specific needs of diverse consumer populations. For example, texture modulation strategies – such as HIU-induced protein dispersion and emulsion stabilization – enable the development of soft, easy-to-swallow gels suitable for elderly individuals with dysphagia. Simultaneously, the ability of HIU, HPP and MW heating to compensate for reduced salt offers a viable pathway for producing low-salt surimi products that meet the dietary requirements of hypertensive patients without compromising palatability. Furthermore, the present review introduces the concept of multi-functional fortification, demonstrating how nutrients such as Ca^2+^, lipids, and polysaccharides can be incorporated into surimi using physical field technologies to achieve dual benefits: enhancing nutritional value while contributing to gel structure formation. This represents a pivotal step toward personalized nutrition through tailored food design.

## CRediT authorship contribution statement

**Xia Gao:** Writing – original draft, Investigation, Funding acquisition, Conceptualization. **Meng Gui:** Conceptualization, Writing – original draft. **Liang Gao:** Investigation. **Jie Huang:** Validation. **Xiaoqing Ren:** Writing – review & editing. **Ru Liu:** Writing – review & editing, Supervision.

## Declaration of competing interest

The authors declare that they have no known competing financial interests or personal relationships that could have appeared to influence the work reported in this paper.

## Data Availability

Data will be made available on request.
